# Telomere Shortening and Accelerated Aging in US Military Veterans

**DOI:** 10.3390/ijerph18041743

**Published:** 2021-02-11

**Authors:** Jeffrey T. Howard, Jud C. Janak, Alexis R. Santos-Lozada, Sarah McEvilla, Stephanie D. Ansley, Lauren E. Walker, Avron Spiro, Ian J. Stewart

**Affiliations:** 1 Department of Public Health, University of Texas at San Antonio, One UTSA Circle, San Antonio, TX 78249, USA; sarahmc.advocare@gmail.com (S.M.); deleons3@livemail.uthscsa.edu (S.D.A.); 2 Consequences of Trauma Working Group, the Center for Community-Based and Applied Health Research, University of Texas at San Antonio, One UTSA Circle, San Antonio, TX 78249, USA; 3 Bexar Data LLC, San Antonio, TX 78210, USA; judjanak@bexardata.org; 4 Department of Human Development and Family Studies, Pennsylvania State University, 119 Health and Human Development Building, University Park, PA 16802, USA; ars39@psu.edu; 5 David Grant USAF Medical Center, Travis Air Force Base, Fairfield, CA 94535, USA; LWalker@themccgroup.com; 6 Massachusetts Veterans Epidemiology Research and Information Center, VA Boston Healthcare System, Boston, MA 02130, USA; aspiro3@bu.edu; 7 Departments of Epidemiology and Psychiatry, Boston University Schools of Public Health and Medicine, Boston, MA 02118, USA; 8 Uniformed Services University of Health Sciences, Bethesda, MD 20814, USA; ian.stewart@usuhs.edu

**Keywords:** accelerated aging, telomeres, veteran’s health

## Abstract

A growing body of literature on military personnel and veterans’ health suggests that prior military service may be associated with exposures that increase the risk of cardiovascular disease (CVD), which may differ by race/ethnicity. This study examined the hypothesis that differential telomere shortening, a measure of cellular aging, by race/ethnicity may explain prior findings of differential CVD risk in racial/ethnic groups with military service. Data from the first two continuous waves of the National Health and Nutrition Examination Survey (NHANES), administered from 1999–2002 were analyzed. Mean telomere length in base pairs was analyzed with multivariable adjusted linear regression with complex sample design, stratified by sex. The unadjusted mean telomere length was 225.8 base shorter for individuals with prior military service. The mean telomere length for men was 47.2 (95% CI: −92.9, −1.5; *p* < 0.05) base pairs shorter for men with military service after adjustment for demographic, socioeconomic, and behavioral variables, but did not differ significantly in women with and without prior military service. The interaction between military service and race/ethnicity was not significant for men or women. The results suggest that military service may contribute to accelerated aging as a result of health damaging exposures, such as combat, injury, and environmental contaminants, though other unmeasured confounders could also potentially explain the results.

## 1. Introduction

Cardiovascular disease (CVD) is the leading cause of death in the United States (US) among both civilians [1] and US military veterans [2]. A growing body of literature suggests that US military veterans, especially those exposed to combat [3,4], have elevated risk of hypertension [4,5] and CVD [3,6,7]. Military veterans may be at risk for CVD due to a variety of factors, including behavioral [8], and exposure to stressful environments, such as combat [3,4] and traumatic injury [6,9], leading to post-traumatic stress disorder (PTSD) [5,10,11,12] and depression [13,14]. Despite the fact that non-Hispanic Blacks in the US tend to have higher CVD risks than non-Hispanic Whites [15,16], a recent study also found that, not only were military veterans at higher risk for CVD overall, but that the risk differed by race/ethnicity such that non-Hispanic White veterans had higher CVD risk compared to non-Hispanic Blacks [7]. Higher income, increased opportunities for physical activity, and lower levels of obesity among non-Hispanic Black veterans relative to non-Hispanic Black non-veterans appeared to partially explain the differences in CVD risk; however, this curious finding raises questions about the underlying physiological mechanisms that might be operating to produce a differential racial/ethnic association between military service and CVD.

One explanation may lie in the relationship between leukocyte telomere length, race/ethnicity, and CVD. Telomeres are the protective end caps on chromosomes that are made of repeating base pairs [17]. As cells divide the telomeres get progressively shorter, eventually leading to cellular senescence [17,18]. Stress exposure [19,20,21] and inflammation [22,23] have both been linked to differential shortening of telomeres, which, in turn, has been linked to increased risk of CVD [24,25]. At the same time, some studies have shown that individuals of recent African descent, including non-Hispanic Blacks living in the US, have longer mean telomere lengths than individuals of recent European descent, despite having similar telomere lengths at birth [26,27,28]. Similarly, while telomere length does not differ for males and females at birth, the rate of telomere shortening over the lifespan does differ by sex, with females tending to have a slower rate of telomere shortening compared to males [29]. Additionally, there is some evidence that military veterans have shorter mean telomere lengths than non-veterans [30,31,32,33], but it is not clear whether the longer mean telomere length in non-Hispanic Black military veterans is associated with lower CVD risks compared to non-Hispanic White military veterans.

The goal of this study was to assess whether there is an association between prior military service and telomere length for males and females, and, if so, if the association differs by race/ethnicity. The primary hypothesis was that individuals with prior military service would have shorter mean telomere length than individuals with no prior military service. The secondary hypothesis was that the military service-telomere length association would differ by race/ethnicity.

## 2. Materials and Methods

### 2.1. Study Design

Public-use data from the first two continuous waves of the National Health and Nutrition Examination Survey (NHANES), collected from 1999 through 2002, were compiled for this study. These were the most recent data available for telomere length from this dataset. The NHANES study is administered in two-year waves, and data for each wave are de-identified and made publicly available by the National Center for Health Statistics. This project was reviewed by the David Grant USAF Medical Center Institutional Review Board and determined to be research not involving human subjects as defined in 45 CFR 46.104(3)(A).

### 2.2. Participants

The NHANES is an ongoing study administered to nationally representative samples of the non-institutionalized, US population, and includes both children and adults. However, the determination of prior service in the US military is based on a questionnaire that is asked only of individuals of appropriate age, those aged 17 years or older at the time of the initial interview. Thus, the initial study population consisted of individuals 17 years and older. In addition, telomeres were measured for adults aged 20 years and older, with a range of 20 to 85 years of age. Thus, the final analytical sample contained only US adults aged 20 years and older at the time of interview. A total of 21,004 participants were included in the 1999–2002 waves of NHANES, and 13,184 were excluded from the analysis (10,713 [81.3%] were under age 20, 2464 [18.7%] either did not have a measurement for telomeres, 2 [<0.01%] had extreme values for telomeres, and 5 [<0.01%] did not indicate whether they had served in the US military or not).

### 2.3. Measures

The outcome measure for this study was telomere length, measured in the number of base pairs (BP). Blood samples taken from participants were analyzed using the polymerase chain reaction (PCR) method [34,35]. Leukocyte telomere length was measured as the mean length of multiple cell types for each individual participant relative to the standard DNA reference (T/S ratio). Mean T/S ratios were converted to base pairs using the following formula: BP = (3274 + 2413 × T/S)) [36].

Independent variables for this study included demographic, socioeconomic, behavioral, and anthropometric measures. Demographic variables included age (as continuous single year), sex (male [reference] and female), race/ethnicity (non-Hispanic White [reference], non-Hispanic Black, Mexican American, Other Hispanic, or Other), and marital status (Never Married [reference], Living with Partner, Married, Separated, Divorced, Widowed, or Missing). Socioeconomic variables included educational attainment (Less than High School [reference], High School Graduate or Equivalent, Some College, or College Graduate or Higher), and poverty indicator (Below Poverty Line [reference], At or Above Poverty Line, or Missing). Behavioral variables included smoking status, alcohol consumption, and physical activity level. Smoking status was categorized as (Never smoked [reference], Current smoker, Former smoker, or Missing). Alcohol consumption was measured as the frequency of binge and non-binge drinking days in the past year where individuals with no alcohol consumption ever coded as Lifetime Abstainers, individuals who no longer drink alcohol coded as Former Drinkers, individuals with no binge drinking days coded as Light Drinkers (1–52 days, no binge drinking) [reference], Moderate Drinkers (53–156 days, no binge drinking), Frequent Drinkers (157–365 days, no binge drinking), and individuals with binge drinking days coded as Infrequent Binge Drinkers (1–12 days in the past year), and Frequent Binge Drinkers (13–365 days in the past year). Physical activity level was measured as quartiles of activity where quartile 1 (reference) is the bottom 25% of the population with the lowest amount of physical activity in the past month and the quartile 4 is the top 25% of the population with the highest amount of physical activity in the past month. The anthropometric measure was body mass index (BMI) status, coded as (20 ≤ BMI < 25 = Normal Weight [reference], BMI < 20 = Under Weight, 25 ≤ BMI < 30 = Over Weight, 30 ≤ BMI = Obese, or Missing).

### 2.4. Statistical Analysis

The descriptive statistics are reported as mean and standard deviation for continuous variables, and as percentages and standard error for categorical variables. Multivariable adjusted linear regression models were estimated for mean telomere length in base pairs, and the results are reported as regression coefficients, 95% confidence intervals (CI), and *p*-values. Regression models were estimated for the total sample and also stratified by sex due to known sex differences in telomere shortening for males and females [29], and due to the fact that military exposures likely differed by sex for this cohort of military veterans. Regression models were adjusted for demographic, socioeconomic, behavioral, and anthropometric variables, including age, race/ethnicity, educational attainment, income to poverty ratio, marital status, smoking status, alcohol use, BMI categories, physical activity quartiles, and prior service in the US military. Interactions between race/ethnicity and military service, and between age and military service were also tested. All analyses were performed in IBM SPSS Statistics version 27 (Chicago, IL, USA), and were adjusted for complex survey design and population weighting through the use of survey procedures. Incorporating population weights, primary sampling unit and stratum variables into the analyses accounts for differential probability of selection into the sample, as well as non-response bias, making the results generalizable to the US adult population.

## 3. Results

### 3.1. Descriptive Analysis

The analysis contained a total of 7820 adults who were aged 20 years or older and indicated whether they had or had not previously served active duty in the US armed forces. Table 1 reports the descriptive statistics for demographic and socioeconomic variables. The mean telomere length was 5821.7, and the unadjusted difference in telomere length between individuals with prior military service and no prior service was 225.8 base pairs. The mean age of the sample was 46.1 years, which differed significantly between individuals with and without prior military service. Individuals with prior military service had a mean age of 55.8 years, while individuals with no prior military service had a mean age of 44.4 years. Individuals with prior service were also more likely to be male (94.4%) than individuals without prior service (44.4%). Race/ethnicity, education, marital status, and income to poverty ratio all differed significantly between individuals with and without prior military service. Individuals with prior military service had a higher percentage who were non-Hispanic White, had a college degree or more, were married, and had incomes at least 4 times the poverty line.

Behavioral and anthropometric variables also differed significantly by prior military service status (Table 2). The percentage of prior smoking (42.8 vs. 22.4) and drinking (23.7 vs. 15.0) behavior was higher among individuals with prior military service, as was current infrequent (16.3 vs. 15.3) and frequent (13.7 vs. 11.6) binge drinking behavior. Individuals with prior military service also had higher percentage of overweight BMI status (42.9 vs. 32.5) and a higher percentage who were in the top quartile for physical activity (20.9 vs. 15.1).

### 3.2. Multivariate Analysis

**Figure 1:**
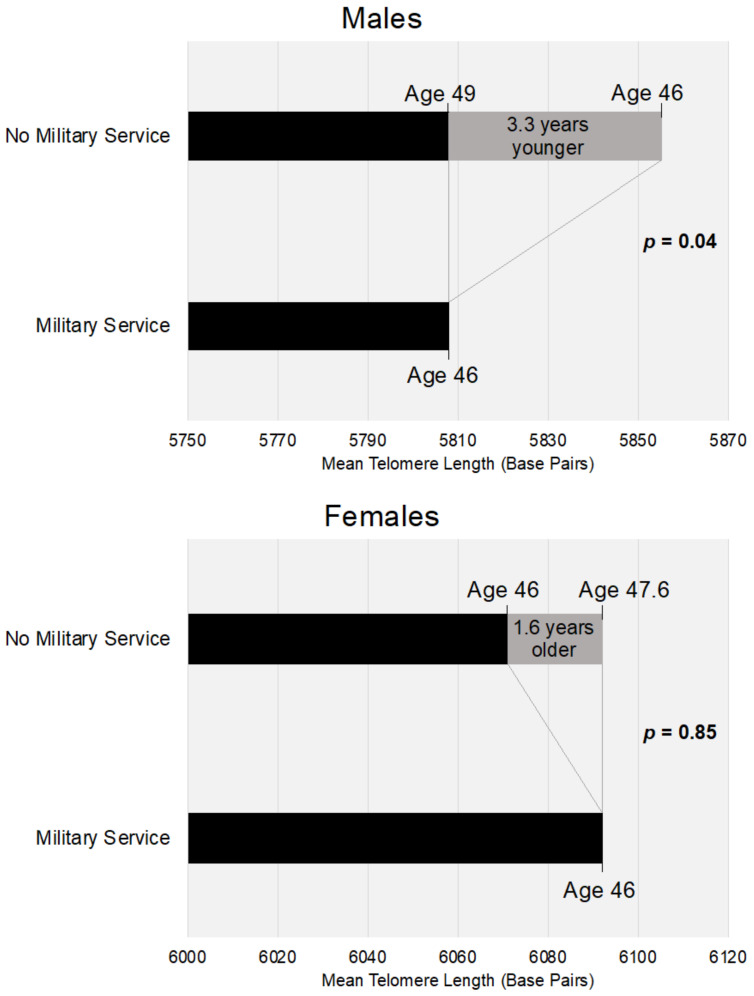
Multivariable regression-based mean telomere length in base pairs for males and females at mean sample age of 46 years, and mean values of covariates (black bars), with year equivalents of telomere difference between individuals with no military service (gray section of bar) compared to individuals with military service.

In multivariable regression models adjusting for demographic, socioeconomic, and behavioral factors, mean telomere length was 43.7 (95% CI: −90.1, 2.7; *p* = 0.06) base pairs shorter for individuals with prior military service compared with individuals with no prior military service, in the total sample. In sex-stratified models, mean telomere length was 47.2 (95% CI: −92.9, −1.5; *p* = 0.04) base pairs shorter for men with prior military service, but mean telomere length did not differ between individuals with and without prior military service among women (Table 3). Each year of age was associated with 14.4 (95% CI: −16.1, −12.6; *p* < 0.001) fewer base pairs for men, and 13.2 (95% CI: −14.9, −11.4; *p* < 0.001) base pairs for women. Dividing the difference in base pairs between individuals with and without prior military service by the mean change in telomere length yields an estimate of the difference in cellular age between individuals with and without prior military service. Therefore, men with no prior military service are, on average, 3.3 years younger in terms of cellular age than similar males with prior military service (*p* < 0.05) (Figure 1). For example, men with prior military service at age 46 have the same mean telomere length as men with no military service do at age 49.

For non-Hispanic Black women, the mean telomere length was 161.8 (95%. CI: 61.9, 261.7; *p* < 0.01) base pairs longer than non-Hispanic White women. There were no other associations between race/ethnicity and telomere length for men or women.

Educational attainment was associated with a significantly longer mean telomere length for men, but not for women. Men with a college degree or more education had telomeres that were 113.2 (95% CI: 41.5, 184.9; *p* < 0.01) base pairs longer than men with less than a high school education.

Men for whom educational attainment was missing had 359.7 (95% CI: −650.8, −68.6; *p* < 0.05) fewer base pairs than men with less than a high school education. Men who were married or separated had mean telomere lengths that were 84.1 (95% CI: −143.7, −24.5; *p* < 0.01) and 147.6 (95% CI: −253.8, −41.5; *p* < 0.01) base pairs shorter, respectively, compared with men who were never married. Women who lived with their partner or widowed had mean telomere lengths that were 187.3 (95% CI: −308.0, −66.5; *p* < 0.01) and 144.0 (95% CI: −256.9, −31.0; *p* < 0.05) base pairs shorter, respectively, compared with women who were never married.

Men for whom alcohol consumption data were missing and women who were frequent drinkers (drink more than once per week but no binge drinking) had mean telomere lengths that were 154.9 (95% CI: 29.8, 280.1; *p* < 0.05) and 71.3 (95% CI: 1.7, 140.9; *p* < 0.05) base pairs longer, respectively, than men and women who were light drinkers (one drinking day per month or less and no binge drinking). Both men and women classified as obese (BMI > 30), had mean telomere lengths that were 88 (95% CI: −159.0, −17.1; *p* < 0.05) and 77.2 (95% CI: −141.4, −12.9; *p* < 0.05) base pairs shorter, respectively, than men and women who were considered normal weight status. Men with higher physical activity levels, specifically those in the top quartile of physical activity, had mean telomere lengths that were 61.2 (95% CI: 7.6, 114.8; *p* < 0.05) base pairs longer than men in the lowest quartile of physical activity. Physical activity level was not associated with telomere length in women. Income to poverty ratio and smoking status were not associated with telomere length for men or women. The interactions between military service and race/ethnicity, and between military service and age were tested in models for men and women, and in all cases the interactions were not statistically significant.

## 4. Discussion

In this study, we found that mean telomere length in base pairs was significantly shorter in men with prior military service compared to men without prior military service, but not in women. We also found that adjusting for potential confounders in multivariable models partially attenuated the difference in mean telomere length between men with and without prior military service, but not completely. The unadjusted difference in mean telomere length between men with and without military service was 225.8 base pairs, while the estimated difference in the model adjusting for demographic, socioeconomic, and behavioral covariates was 47.2 base pairs. Thus, the association between military service and mean telomere length was attenuated by 79% but remained statistically significant after adjustment. The remaining amount of telomere shortening (47.2 base pairs) was equivalent to 3.3 years of additional aging, all other things being equal. These results suggest that, in men, significant residual telomere shortening is associated with prior military service, over and above variability explained by differences in demographic, socioeconomic, and behavioral profiles. While we did find that mean telomere length was significantly longer in non-Hispanic Black women compared to non-Hispanic White women, we found no evidence that prior military service was associated with shorter mean telomere length among women. We also found no evidence for our secondary hypothesis that the association between military service and shorter mean telomere length in men differed by race/ethnicity.

One potential explanation for the differential telomere shortening among men with prior military service is the potential for repeated exposure to highly stressful environments. Military service has many inherently stressful exposures, such as deployments away from friends and family, high physical and mental demands, and the possibility of combat exposure. Chronic and high-intensity stress exposure has been linked to many health problems, including PTSD [4] and hypertension [4], as well as telomere shortening [20,21]. Stress exposure has also been implicated in telomere shortening among military veterans with combat exposure [32], PTSD [31,32] and issues with hostility [33]. The fact that telomere shortening was observed for men, but not women, in our sample may further support the stress hypothesis since women were not permitted in combat roles in the US military until 2013, and thus would have been less likely to be exposed to similar levels of combat stress as male counterparts during the time they served. However, women are disproportionately at risk for other traumatic events in the military such as sexual trauma [37].

Military service may also lead to physiological dysregulation arising from exposures to (1) physical injuries, both combat and non-combat, and (2) other environmental toxins. Combat injuries have been linked to development of PTSD [4,5,12], hypertension [4,5,6], sleep disruptions [4], and inflammation [38,39], all of which have been associated with telomere shortening [23,31,32,40,41,42]. Similarly, military service has been associated with increased risk of exposure to many environmental toxins [43,44], including smoke inhalation, chemical weapons, and water and air pollution, which have also been associated with telomere shortening [45,46,47,48]. Specifically, the mean age for military veterans in this sample is 56 years, which would mean this cohort would have been largely active during the Vietnam War. Significant levels of PTSD [49] and toxin exposure, such as Agent Orange [50,51], are known issues within the Vietnam cohort. Thus, these exposures could explain the associations between military service and telomere shortening in this study. While not universally detrimental, military service may pose a multitude of occupational and environmental risks that ultimately work to accelerate the aging process by increasing stress, inflammation, mood disorders and depression, and hypertension, which become expressed through more rapid shortening of telomeres and other biological markers of aging [52,53].

### Strengths and Limitations

The main strength of this study is the use of data from nationally representative surveys, with many demographic and socioeconomic factors, as well as biological measures of telomere length. This allows for multivariable adjustment of many important demographic and socioeconomic risk factors. Another strength is the relatively large sample size. However, the study does have some limitations. First, the study is observational in nature, which limits causal inference. Also, while we have attempted to control for important correlates and confounders of telomere length, it is not possible to account for every possible risk factor and confounder, therefore, residual confounding is still possible. Specifically, while we adjusted for age differences in our multivariable regression models, it is still possible that residual age-confounding could exist and bias the results. Additionally, the NHANES only collected data on telomere length during the first two waves of the continuous study, from 1999–2002. Given the ages of the military sample, many of the military veterans in this study would have likely been serving active duty during the Vietnam War, but it does not contain data for military veterans of the most recent conflicts in Afghanistan and Iraq. Thus, the observed telomere shortening for military veterans could be attributable to exposures that were unique to the Vietnam setting. Similarly, the NHANES data do not contain longitudinal measures of telomere length over time, so changes in telomere length over time cannot be assessed. Additionally, the method of measuring telomere length in these individuals involves quantifying the mean length of telomeres across many cells and cell types, which could obscure shortening that may be associated with specific cell types. While NHANES did collect data on depression, anxiety, and panic disorders, the diagnostic interviews were only administered to a small subset of participants, therefore, data on mental health disorders were not included in this analysis. Finally, while military service has the potential to involve exposure to highly stressful environments, NHANES does not contain data on specific stress exposures, such as number of deployments, exposure to combat, and exposure to environmental toxins. Thus, the specific types, number, intensity, and combination of exposures during military service, biological mediators, such as inflammatory responses, required to induce accelerated cellular aging through telomere shortening remains unclear.

## 5. Conclusions

Based on data from the 1999–2002 NHANES study, our analysis supports the hypothesis that prior military service is associated with significant telomere shortening in men, but not women. The results add to the growing literature regarding the potential for repetitive exposure to highly stressful occupational environments to accelerate the cellular aging process. More research is needed to provide more clarity around the number and intensity of exposures to stressful environments required, and to understand in more detail the physiological pathways, including inflammatory processes, through which military service operates to shorten telomeres.

## Figures and Tables

**Table 1:** Weighted demographic and socioeconomic characteristics (*n* = 7820).

Variables	Total Sample	Prior Military Service	No Prior Military Service	*p* Value *
Telomere length base pairs, mean (SE)	5821.7 (35.4)	5630.0 (30.0)	5855.8 (37.6)	<0.001
Age in years, mean (SE)	46.1 (0.4)	55.8 (0.6)	44.4 (0.4)	<0.001
Male Sex, percent (SE)	48.6 (0.5)	94.4 (0.8)	40.5 (0.7)	<0.001
Race/Ethnicity, percent (SE)				<0.001
Non-Hispanic White	72.9 (1.8)	84.8 (1.5)	70.8 (2.0)	
Non-Hispanic Black	9.3 (1.1)	8.2 (0.9)	9.5 (1.1)	
Mexican American	7.0 (0.9)	2.2 (0.4)	7.8 (1.0)	
Other Hispanic	6.8 (1.6)	3.3 (1.1)	7.4 (1.8)	
Other	4.0 (0.6)	1.4 (0.4)	4.5 (0.7)	
Education, percent (SE)				<0.001
Less than High School	21.4 (0.9)	14.7 (1.2)	22.6 (0.9)	
High School Graduate or Equivalent	26.0 (1.0)	28.3 (2.1)	25.6 (1.0)	
Some College	28.4 (0.9)	28.6 (1.9)	28.3 (1.0)	
College Degree or More	24.1 (1.6)	28.3 (2.1)	23.4 (1.6)	
Missing	0.1 (0.0)	0.0 (0.0)	0.1 (0.1)	
Marital Status				<0.001
Never Married	15.7 (0.8)	8.6 (1.1)	17.0 (0.9)	
Married	56.4 (1.4)	69.1 (2.1)	54.2 (1.4)	
Live with Partner	5.3 (0.6)	3.3 (0.4)	5.7 (0.7)	
Widowed	5.9 (0.4)	4.1 (0.6)	6.3 (0.4)	
Separated	2.7 (0.2)	1.4 (0.4)	3.0 (0.3)	
Divorced	8.5 (0.5)	8.2 (1.5)	8.5 (0.5)	
Missing	5.4 (1.6)	5.4 (1.6)	5.4 (1.7)	
Income to Poverty Ratio				<0.001
At or Below Poverty	13.1 (0.9)	5.8 (0.9)	14.4 (0.9)	
1.0 to 2.0 Times Poverty	18.9 (1.2)	15.9 (1.6)	19.4 (1.2)	
2.01 to 3.0 Times Poverty	14.4 (0.6)	15.7 (1.3)	14.1 (0.6)	
3.01 to 4.0 Times Poverty	12.7 (0.6)	13.8 (1.1)	12.5 (0.7)	
More than 4.0 Times Poverty	33.3 (1.6)	41.7 (2.0)	31.8 (1.6)	
Missing	7.6 (0.8)	7.0 (1.1)	7.8 (0.9)	
Telomere length base pairs, mean (SE)	5821.7 (35.4)	5630.0 (30.0)	5855.8 (37.6)	<0.001

* Rao-Scott Chi-Square Test.

**Table 2:** Weighted behavioral and anthropometric characteristics (*n* = 7820).

Variables	Total Sample (*n* = 7820)	Prior Military Service (*n* = 1225)	No Prior Military Service (*n* = 6595)	*p* Value *
Smoking Status, percent (SE)				<0.001
Current Smoker	24.4 (0.9)	23.8 (1.5)	24.5 (1.1)	
Former Smoker	25.5 (0.9)	42.8 (1.5)	22.4 (0.9)	
Never Smoker	50.0 (1.3)	33.4 (1.4)	52.9 (1.3)	
Missing	0.1 (0.04)	0.03 (0.03)	0.1 (0.05)	
Alcohol Consumption, percent (SE)				<0.001
Lifetime Abstainer	12.2 (1.6)	4.5 (0.8)	13.5 (1.8)	
Former Drinkers	16.3 (0.8)	23.7 (1.5)	15.0 (0.9)	
Light Drinkers (no binge drinking)	30.5 (1.0)	26.3 (1.9)	31.3 (1.2)	
Moderate Drinkers (no binge drinking)	2.8 (0.3)	2.9 (0.6)	2.8 (0.3)	
Frequent Drinkers (no binge drinking)	5.8 (0.5)	9.6 (1.2)	11.6 (0.6)	
Infrequent Binge Drinker	15.4 (0.7)	16.3 (1.4)	15.3 (0.7)	
Frequent Binge Drinker	11.9 (0.5)	13.7 (1.1)	11.6 (0.6)	
Missing	5.1 (0.4)	3.0 (0.6)	5.5 (0.5)	
Body Mass Index, percent (SE)				<0.001
Underweight	5.4 (0.3)	1.9 (0.3)	6.0 (0.3)	
Normal weight	28.4 (0.9)	22.8 (1.7)	29.4 (1.0)	
Overweight	34.1 (0.9)	42.9 (2.1)	32.5 (1.0)	
Obese	29.6 (1.0)	30.2 (1.3)	29.5 (1.1)	
Missing	2.5 (0.3)	2.3 (0.5)	2.5 (0.3)	
Physical Activity Level Quartiles, percent (SE)				
Quartile 1	14.9 (0.7)	13.7 (1.1)	15.1 (0.9)	0.006
Quartile 2	14.6 (0.7)	13.1 (1.1)	14.8 (0.7)	
Quartile 3	17.1 (0.8)	17.8 (1.6)	16.9 (0.9)	
Quartile 4	15.9 (1.0)	20.9 (2.2)	15.1 (0.9)	
Missing	37.5 (1.3)	34.5 (1.7)	38.0 (1.4)	

* Rao-Scott Chi-Square Test.

**Table 3:** Weighted results of total sample and sex-stratified, multivariable linear regression analyses of telomere length in base pairs (*n* = 7820).

Variables	Total (*n* = 7820) Est (95% CI)	Males (*n* = 3766) Est (95% CI)	Females (*n* = 4054) Est (95% CI)
Intercept	6555.5 (6343.1, 6767.8) ***	6455.9 (6261.7, 6650.1) ***	6606.8 (6356.1, 6857.5) ***
Military Service			
Military vs. No Military (ref)	−43.7 (−90.1, 2.7)	−47.2 (−92.9, −1.5) *	21.4 (−211.3, 254.0)
Age in years	−13.7 (−15.1, −12.2) ***	−14.4 (−16.1, −12.6) ***	−13.2 (−14.9, −11.4) ***
Sex, Males vs. Females (ref)	−30.6 (−62.6, 1.5)	n/a	n/a
Race/Ethnicity			
Non-Hispanic White (ref)			
Non-Hispanic Black	114.2 (33.1, 195.3) **	63.8 (−18.0, 145.5)	161.8 (61.9, 261.7) **
Mexican American	−98.2 (−208.6, 12.2)	−93.2 (−208.9, 22.5)	−104.3 (−223.8, 15.2)
Other Hispanic	94.7 (−112.1, 301.4)	68.4 (−129.8, 266.6)	119.2 (−106.3, 344.6)
Other	−38.9 (−151.0, 73.2)	−57.0 (−188.4, 74.4)	−25.6 (−163.3, 112.1)
Education			
Less than High School (ref)			
High School Graduate or Equivalent	55.3 (0.5, 110.1) *	41.2 (−24.5, 106.9)	66.3 (−15.9, 148.4)
Some College	67.1 (6.0, 128.2) *	54.0 (−1.3, 109.4)	84.0 (−27.8, 195.8)
College Degree or More	97.8 (31.0, 164.6) **	113.2 (41.5, 184.9) **	81.5 (−28.3, 191.3)
Missing	−22.5 (−594.2, 549.3)	−359.7 (−650.8, −68.6)*	147.7 (−738.7, 1034.1)
Marital Status			
Never Married (ref)			
Married	−81.6 (−129.8, −33.4) **	−84.1 (−143.7, −24.5) **	−76.4 (−153.4, 0.7)
Live with Partner	−44.9 (−96.8, 7.0)	−56.0 (−177.4, 65.5)	−187.3 (−308.0, −66.5) **
Widowed	−114.5 (−209.2, −19.7) *	−110.0 (−238.6, 18.8)	−42.0 (−127.5, 43.4)
Separated	−119.8 (−199.9, −30.8) *	−123.3 (−275.8, 29.1)	−144.0 (−256.9, −31.0) *
Divorced	−126.4 (−222.3, −30.6) *	−147.6 (−253.8, −41.5) **	−104.2 (−219.1, 10.7)
Missing	153.9 (12.7, 295.0) *	137.0 (−13.2, 287.3)	165.4 (−38.9, 369.7)
Income to Poverty Ratio			
At or Below Poverty (ref)			
1.0 to 2.0 Times Poverty	−83.1 (−190.1, 23.8)	−35.6 (−142.7, 71.4)	−115.2 (−243.5, 13.2)
2.01 to 3.0 Times Poverty	−48.3 (−158.9, 62.3)	−11.3 (−154.0, 131.3)	−74.8 (−202.1, 52.5)
3.01 to 4.0 Times Poverty	−32.9 (−163.6, 97.8)	46.6 (−106.0, 199.1)	−100.9 (−232.2, 30.4)
>4.0 Times Poverty	−47.3 (−113.2, 142.3)	21.1 (−96.7, 174.3)	−101.6 (−249.1, 46.0)
Missing	14.5 (−113.2, 142.3)	61.0 (−52.3, 174.3)	−18.7 (−187.0, 149.6)
Smoking Status			
Never Smoker (ref)			
Current Smoker	−4.3 (−44.3, 35.7)	36.2 (−17.8, 90.3)	−43.5 (−108.4, 21.5)
Former Smoker	−34.0 (−82.0, 14.0)	−11.4 (−74.3, 51.4)	−50.7 (−114.4, 12.7)
Missing	99.7 (−175.4, 374.7)	271.3 (−130.8, 673.4)	24.5 (−364.3, 413.3)
Alcohol Consumption, percent			
Lifetime Abstainer	41.6 (−40.6, 123.9)	53.3 (−85.6, 192.2)	20.4 (−67.3, 108.0)
Former Drinkers	−35.6 (−88.0, 16.7)	−10.2 (−107.9, 87.6)	−59.9 (−130.6, 10.8)
Light Drinkers (no binge drinking) (ref)			
Moderate Drinkers (no binge drinking)	−39.6 (−99.3, 20.1)	26.4 (−78.8, 131.4)	−108.1 (−235.4, 19.2)
Frequent Drinkers (no binge drinking)	58.8 (4.9, 112.7) *	59.1 (−43.9, 162.2)	71.3 (1.7, 140.9) *
Infrequent Binge Drinker	15.0 (−45.6, 75.6)	17.1 (−80.3, 114.4)	17.5 (−93.0, 128.1)
Frequent Binge Drinker	14.4 (−41.4, 70.2)	41.9 (−29.7, 113.5)	−54.9 (−180.2, 70.4)
Missing	98.1 (16.8, 179.3) *	154.9 (29.8, 280.1) *	51.1 (−44.6, 146.9)
Body Mass Index			
Normal weight (ref)			
Underweight	−16.9 (−131.9, 98.1)	−30.6 (−165.1, 104.0)	6.2 (−132.1, 144.4)
Overweight	−53.8 (−116.2, 8.6)	−52.8 (−127.9, 22.2)	−49.9 (−138.3, 18.5)
Obese	−77.9 (−132.7, −23.1) **	−88.0 (−159.0, −17.1) *	−77.2 (−141.4, −12.9) *
Missing	−8.3 (−96.2, 79.7)	154.9 (29.8, 280.1) *	44.4 (−80.5, 169.3)
Physical Activity Level Quartiles			
Quartile 1 (ref)			
Quartile 2	−45.9 (−116.2, 8.6)	0.2 (−76.7, 77.2)	−87.0 (−203.2, 29.2)
Quartile 3	−5.0 (−84.8, 74.8)	15.5 (−56.2, 87.1)	−18.2 (−131.0, 94.7)
Quartile 4	55.3 (−0.7, 111.4)	61.2 (7.6, 114.8) *	68.8 (−43.4, 181.1)
Missing	−14.1 (−68.0, 39.7)	45.3 (−5.4, 96.0)	−64.1 (−150.5, 22.5)

* p ≤ 0.05; ** *p* ≤ 0.01; *** *p* ≤ 0.001. All variables listed in the table are included in the model.

## Data Availability

All data used in this study are publicly available from the U.S. CDC website: https://wwwn.cdc.gov/nchs/nhanes/.
